# Divergent brain regional atrophy and associated fiber disruption in amnestic and non-amnestic MCI

**DOI:** 10.1186/s13195-023-01335-1

**Published:** 2023-11-13

**Authors:** Chao Du, Mingxi Dang, Kewei Chen, Yaojing Chen, Zhanjun Zhang

**Affiliations:** 1 State Key Laboratory of Cognitive Neuroscience and Learning, Beijing Normal University, Beijing, 100875 China; 2 Beijing Aging Brain Rejuvenation Initiative Centre, Beijing Normal University, Beijing, 100875 China; 3 Research Institute of Intelligent and Complex Systems, Fudan University, Shanghai, 200433 China; 4 Banner Alzheimer’s Institute, Phoenix, AZ 85006 USA; 5 Arizona State University, Temple, AZ 85281 USA

**Keywords:** Amnestic and non-amnestic mild cognitive impairment, Hippocampus, Inferior frontal gyrus, Volume atrophy, White matter fiber bundles

## Abstract

**Background:**

Understanding the pathological characteristics of various mild cognitive impairment (MCI) subtypes is crucial for the differential diagnosis of dementia. The purpose of this study was to feature divergent symptom-deficit profiles in amnestic MCI (aMCI) and non-amnestic MCI (naMCI).

**Methods:**

T1 and DTI MRI data from a total of 158 older adults with 50 normal controls, 56 aMCI, and 52 naMCI were included. The voxel-wise gray matter volumes and the number of seed-based white matter fiber bundles were compared among these three groups. Furthermore, correlation and mediation analyses between the neuroimaging indices and cognitive measures were performed.

**Results:**

The aMCI with specific memory abnormalities was characterized by volumetric atrophy of the left hippocampus but not by damage in the linked white matter fiber bundles. Conversely, naMCI was characterized by both the altered volume of the right inferior frontal gyrus and the significant damage to fiber bundles traversing the region in all three directions, not only affecting fibers around the atrophied area but also distant fibers. Mediation analyses of gray matter-white matter-cognition showed that gray matter atrophy affects the number of fiber bundles and further affects attention and executive function. Meanwhile, fiber bundle damage also affects gray matter volume, which further affects visual processing and language.

**Conclusions:**

The divergent structural damage patterns of the MCI subtypes and cognitive dysfunctions highlight the importance of detailed differential diagnoses in the early stages of pathological neurodegenerative diseases to deepen the understanding of dementia subtypes and inform targeted early clinical interventions.

**Supplementary Information:**

The online version contains supplementary material available at 10.1186/s13195-023-01335-1.

## Introduction

The heterogeneity among the patients may be the cause of the failure of clinical intervention studies targeting mild cognitive impairment (MCI), the early stage of dementia [1]. Based on the differences of cognitive impairments, MCI can be classified into amnestic MCI (aMCI; [2]) or non-amnestic MCI (naMCI; [3]), which primarily leads to Alzheimer’s disease (AD; [2]) and frontotemporal dementia (FTD; [4]) respectively. The brain damage exhibited in AD mainly occurs in the medial temporal lobe, particularly the hippocampus and entorhinal cortex. Conversely, FTD is distinguished from AD by frontal lobe damage [5]. Investigating the pathologic characteristics of these two MCI subtypes is crucial for successful early intervention efforts and differential diagnosis of dementia.

The loss of neurofibrils and neuronal soma are two distinct pathologic processes that occur in different degrees and locations in AD and FTD. Researches have shown that white matter atrophy plays an important role in FTD pathophysiology [6], distinct from the predominant gray matter damage observed in AD [7]. This difference in affected areas and tissues has been linked to specific cognitive impairments in these dementias. For instance, executive dysfunction in FTD is related to both gray and white matter damage in the frontal lobe, while memory decline in AD is associated with gray matter damage in the temporal lobe [7]. Another recent study found that alterations to short-range white matter fiber bundles impacted language, while alterations to long-range white matter fiber bundles affected executive function [8]. The MCI population exhibits extensive cognitive impairment, making them an ideal choice for exploring patterns of brain structure damage that may reflect distinct neuropathological aging processes.

In the present study, we aim to clarify the characteristics of key brain structural pathologic damages in patients with aMCI and naMCI. To accomplish this, we employed voxel-based morphometric analysis of gray matter and seed-based individual white matter bundle deterministic fiber tracking. We hypothesized that (1)  both aMCI and naMCI would exhibit unique gray matter damage patterns, with naMCI showing more extensive white matter injury; (2) white matter connectivity damage in individuals with naMCI is not only present in the proximal regions but also in the distal regions, reflecting differential cognitive impairments; and that (3) cognitive functions requiring coordination among multiple brain regions, such as attention and execution, would have a closer association with white fiber connectivity, whereas other cognitive functions may be more closely related to the volume of local brain regions.

## Materials and methods

### Participants

This study included 158 native Chinese participants, all from an ongoing longitudinal study that collected comprehensive brain imaging and neurocognitive test data in an elderly community-dwelling sample, the Beijing Aging Brain Rejuvenation Initiative (BABRI) [9], which comprises a large database of 9532 records. The participant inclusion criteria were as follows: (1) more than 6 years of education; (2) aged between 50 and 80 years old; (3) right-handed; (4) scored no less than 24 on the Mini-Mental Status Examination (MMSE), Chinese version; (5) no history of nephritis, coronary disease, gastrointestinal disease, tumors, psychiatric illness, or dementia; (6) no contraindications to MRI; (7) availability of complete neuropsychological tests for MCI assessment. The Ethics Committee and Institutional Review Board of Beijing Normal University’s Imaging Centre for Brain Research approved this study (ICBIR_A_0041_002.02), and all participants provided written informed consent. The MRI data from his/her first visit for each participant were selected for the current analysis together with the neuropsychological test measures. The flowchart of participant selection is shown in Supplemental Fig. 1.

### Neuropsychological testing

Each participant completed a series of neuropsychological tests, including the MMSE [10] and five other tests of cognitive domains: (1) memory, delay, and total part of the Auditory Verbal Learning Test (AVLT) [11] and the Rey-Osterrieth Complex Figure (R-O) test-recall [12]; (2) visual ability, the Rey-Osterrieth Complex Figure (R-O) test-copy and the Clock-Drawing Test (CDT) [13]; (3) language, the Category Verbal Fluency Test (CVFT) [14] and the Boston Naming Test (BNT) [15]; (4) attention, the Symbol Digit Modalities Test (SDMT) [16] and Part A of the Trail-Making Test (TMTA) [17]; (5) execution, time index of Part C of the Stroop Test [18] and Part B of the Trail-Making Test (TMTB).

The diagnosis of MCI was made following Petersen’s criteria [19] and was performed by expert neurologist Z.J.Z. with 27 years of experience in clinical neurology and Y.J.C., a researcher with 12 years of research experience in the field, both blinded to the results of MRI. The validation of the diagnosis was performed by D.C. at the time of subject inclusion. The accuracy of the diagnosis was ensured by evaluating each cognitive domain with 2–3 cognitive tests. Impairment in a specific domain was determined if the participant had at least two cognitive test scores that were more than 1.5 standard deviations below the age- and education-adjusted norm. Specifically, aMCI was diagnosed based on memory impairment, while naMCI was diagnosed based on intact memory and the impairment of at least one other cognitive domain (i.e., visual ability, language, attention, and/or executive function). Drawing upon the aforementioned criteria, we classified the baseline data of 5990 individuals with assessable MCI into the following categories: 4822 individuals exhibited normal cognitive function elderly, 480 were diagnosed with single-domain aMCI, 480 with naMCI, and 208 with multi-domain aMCI (MCI with memory impairment and impairment in one or more additional cognitive domains). Furthermore, within the dataset containing baseline MRI records from 953 individuals, we identified 24 cases of multi-domain aMCI (see Supplemental Fig. 1). The participants included in our final analysis of this current study were aMCI group which consisted of participants with single-domain impairment only, while the naMCI group included participants with single- or multi-domain impairments, aimed at better distinguishing between MCI with and without memory impairment. The normal control group included participants who demonstrated no impairment on all the above cognitive tests, no subjective cognitive complaint, and were matched on demographic variables and numbers with the other groups. The final sample consisted of 50 normal control individuals, 56 aMCI individuals, and 52 naMCI individuals. DTI data from 6 aMCI and 1 naMCI subject were excluded due to quality issues.

### Image data acquisition

Structural magnetic resonance imaging data were collected by a Siemens Trio 3.0 Tesla scanner (Trio; Siemens, Erlangen, Germany) in the Imaging Center for Brain Research at Beijing Normal University. Participants laid supine with their head snugly fixed in place by straps and foam pads to minimize head movement. T1-weighted, sagittal 3D magnetization-prepared rapid gradient echo sequences were acquired of the entire brain [sagittal slices = 176, repetition time (TR) = 1900 ms, echo time (TE) = 3.44 ms, slice thickness = 1 mm, flip angle = 9°, inversion time = 900 ms, field of view (FOV) = 256 × 256 mm^2^, acquisition matrix = 256 × 256]. Each participant underwent two sets of DTI sequences with the following scan parameters: TR = 9500 ms; TE = 92 ms; 30 diffusion-weighted directions with a b-value of 1000 s/mm^2^, and a single image with a b-value of 0 s/mm^2^; slice thickness = 2 mm; no inter-slice gap; 70 axial slices; matrix size = 128 × 128; field of view (FOV) = 256 × 256 mm^2^; and voxel size = 2 × 2 × 2 mm^3^.

### Image data processing

T1 imaging data was pre-processed using Statistical Parametric Mapping 12 (SPM12: www.fil.ion.ucl.ac.uk/spm) via MATLAB R2012b (MathWorks Inc., MA). The CAT toolbox within SPM12 was utilized to perform voxel-based morphometry analysis [20]. The images were skull-stripped and bias-corrected and segmented into gray matter, white matter, or cerebrospinal fluid maps which were normalized with the DARTEL algorithm and smoothed with an 8-mm full-width half-maximum Gaussian kernel. Total intracranial volume (TIV) was computed for each participant by summing the volumes of gray matter, white matter, and cerebrospinal fluid and used as a covariate in the subsequent analysis.

The DTI data were pre-processed using the Pipeline for Analyzing braiN Diffusion imAges (PANDA: www.nitrc.org/projects/panda/) through MATLAB. The data was coregistered to the b0 image using an affine transformation to correct the eddy-current induced distortions and simple head-motion artifacts. Then, the diffusion gradient directions were adjusted, and tensor matrix and diffusion tensor metrics were calculated, with the main focus being fractional anisotropy (FA). A seed-based approach was used for deterministic fiber tracking, with fibers crossing the seed point being included without directional restrictions. The stopping criteria were an FA value of at least 0.2 and a bending angle not exceeding 45° within a voxel.

### Statistical analysis

The voxel-wise gray matter volumes were compared among aMCI, naMCI and control groups using SPM12 after adjusting for age, sex, years of education, and TIV. The significance level was set at a voxel-level *p* < 0.001 with a cluster-level *p* < 0.05 (GRF-corrected). The volumes of significant gray matter clusters were compared between groups, and cluster masks were used in the subsequent white matter analyses.

Comparison of the structural connectivity among groups was based on inseparable local brain volume. The deterministic fiber tracking in each individual DTI space was passed through the seeds from the gray matter analysis. In addition, to explore the change pattern of different directions and distances of fiber bundles, we segmented the tracked fibers with the center point of gray matter region along the three orthogonal directions in the 3D space (X, Y, Z in MNI coordinates) separately on the average value of each participant. The number of fiber bundles and each segment among groups were compared with the significance level at *p* < 0.05 with FDR-corrected over segments.

Correlations between neuroimaging indices and cognitive test scores (corrected for age, gender and education) were computed, with a significant level at *p* < .05 to make these changes of neuroimaging indexes be meaningful and interpretable. Moreover, mediation analyses were performed with gray matter volume and white matter fiber bundle number, both serving as an independent variable and a mediator, and cognitive test scores as the dependent variable. The analyses were performed using bootstrap method to determine which of the local regions’ volume or connectivity is more directly relevant to cognitive functions across all populations in this study.

### Data availability statement

Data and code used in this study can be provided upon request to the authors after establishing a formal data sharing agreement.

## Results

### Demographic characteristics and neuropsychological testing

Demographic and neuropsychological testing results of the control, aMCI, and naMCI groups are presented in Table 1. There were no significant differences in age, gender, or education among the three groups. As expected, both MCI subtypes showed worse cognitive performance than the control group in all domains. Among the two MCI subtypes, naMCI performed better in memory (*F* = 19.6–107.7, *all p* < 0.05), while aMCI performed better in visual ability, attention, and execution (*F* = 18.9–48.6, *all p* < 0.05).

**Table 1:** Demographics of participants

	Controls (*n* = 50)	aMCI (*n* = 56)	naMCI (*n* = 52)	*F/x*^*2*^	*p*
Age (years)	65.0 ± 7.0	66.1 ± 8.0	66.1 ± 8.0	0.3	0.726
Education (years)	11.6 ± 3.0	11.5 ± 3.0	10.8 ± 3.5	0.9	0.418
Male/female	21/29	20/36	18/34	0.7	0.707
MMSE	28.1 ± 1.4	27.0 ± 1.8	26.9 ± 1.7	8.9	< 0.001^a,b^
**Memory**					
AVLT delay	5.7 ± 2.0	1.2 ± 1.1	4.4 ± 1.7	107.7	< 0.001^a,b,c^
AVLT total	30.0 ± 7.0	15.5 ± 4.3	25.5 ± 6.2	85.1	< 0.001^a,b,c^
R-O delay	14.9 ± 4.7	8.1 ± 6.3	10.2 ± 5.9	19.6	< 0.001^a,b,c^
**Visual ability**					
R-O copy	35.0 ± 1.3	32.7 ± 5.5	28.6 ± 7.1	18.9	< 0.001^a,b,c^
CDT	25.0 ± 3.2	22.4 ± 5.2	21.0 ± 6.2	8.3	< 0.001^a,b^
**Language**					
CVFT	46.1 ± 8.2	40.7 ± 9.2	39.9 ± 8.7	7.8	< 0.001^a,b^
BNT	25.0 ± 2.4	22.1 ± 3.5	21.9 ± 3.9	13.4	< 0.001^a,b^
**Attention**					
SDMT	37.5 ± 7.0	31.8 ± 8.3	23.2 ± 6.9	46.4	< 0.001^a,b,c^
TMTA	51.3 ± 12.8	58.4 ± 15.8	96.6 ± 38.6	48.6	< 0.001^b,c^
**Execution**					
StroopCtime	74.4 ± 14.6	89.9 ± 27.9	94.6 ± 28.6	9.3	< 0.001^a,b^
TMTB	139.1 ± 34.8	191.9 ± 66.2	259.5 ± 85.1	42.8	< 0.001^a,b,c^

*aMCI* Amnestic mild cognitive impairment, *naMCI* Non-amnestic mild cognitive impairment, *MMSE* Mini Mental Status Examination, *AVLT* Auditory Verbal Learning Test, *R-O* Rey-Osterrieth Complex Figure, *CDT* Clock Drawing Test; *CVFT*, Category Verbal Fluency Test, *BNT* Boston Naming Test, *SDMT* Symbol Digit Modalities Test, *TMT* Trail Making Test, *StroopCtime* Time of Stroop Word Color Test C

^a^Statistically significant difference between controls and aMCI at *p* < 0.05

^b^Statistically significant difference between controls and naMCI at *p* < 0.05

^c^Statistically significant difference between aMCI and naMCI at *p* < 0.05

### Atrophy and dysfunction of white matter fiber bundles in the two MCI subtypes

**Fig. 1:**
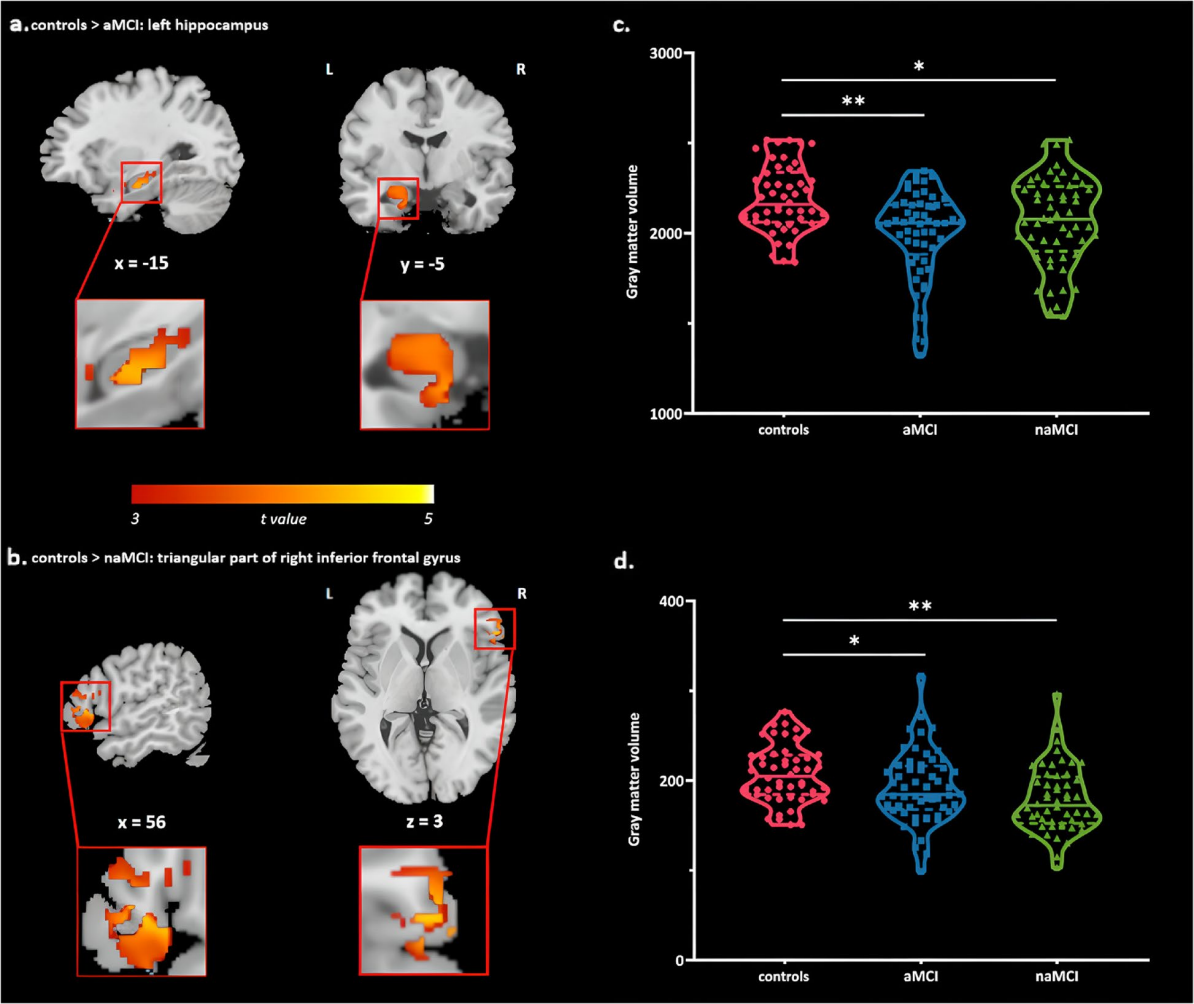
The figure shows the difference in gray matter between groups, with a significance level set at a voxel-level of *p* < 0.001 and a cluster-level of *p* < 0.05 (GRF-corrected). The controlled covariates were age, sex, years of education, TIV. The aMCI group showed significant reduction in gray matter volume (peak locates on the left hippocampus) compared to the control group (**a**); the naMCI group showed significant reduction in gray matter volume (peak locates on the triangular part of right inferior frontal gyrus) compared to control group (**b**); the plots show the differences in the left hippocampus (**c**) and the triangular part of right inferior frontal gyrus (**d**) volume among the groups, respectively

**Fig. 2:**
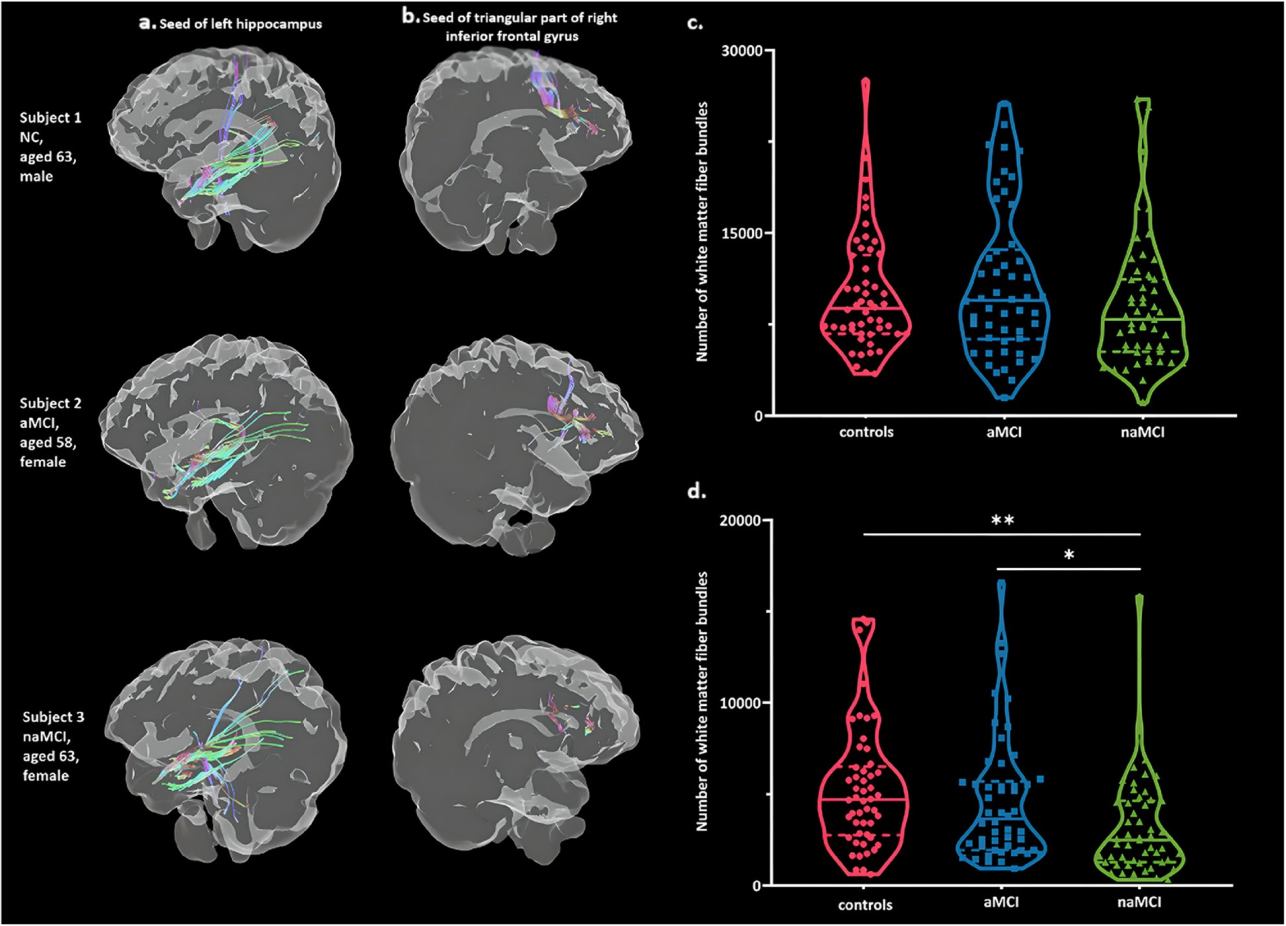
Visualization examples and group differences of fiber bundle number based on gray matter regions. The example of fiber bundles in each group were based on the seed of the left hippocampus (**a**) and the triangular part of right inferior frontal gyrus (**b**), respectively; the plots show the group differences of fiber bundle number based on the seed of the left hippocampus (**c**) and the triangular part of right inferior frontal gyrus (**d**), respectively

aMCI showed atrophy mainly in the left hippocampus compared to controls (Supplemental Table 1 and Fig. 1a), but there was no significant reduction in the number of white matter fiber bundles passing through the left hippocampus (Supplemental Table 2 and Fig. 2a). Further analysis of the fiber bundles at each of the three directions and with different distances showed no significant disruption in aMCI (Supplemental Table 3 and Supplemental Fig. 2).

**Fig. 3:**
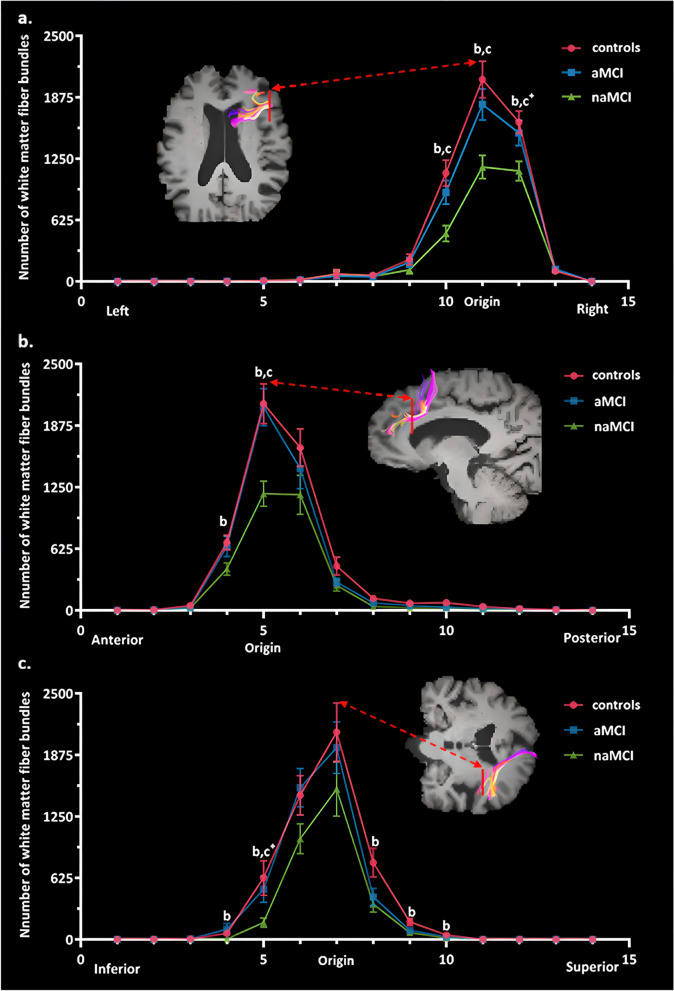
Group differences of each fiber bundle segment number based on the seed of the triangular part of right inferior frontal gyrus along *X*, *Y*, *Z* axis (MNI coordinates). The central point of the seed in the *X*, *Y*, *Z* axis direction fell on the 11th, the 5th, and the 7th segment, separately. The symbol “^b^” indicates the following: statistically significant difference between controls and naMCI at FDR-corrected *p* < 0.05. The symbol “^c^” indicates the following: statistically significant difference between aMCI and naMCI at FDR-corrected *p* < 0.05. The symbol “^c+^” indicates the following: statistically marginally significant difference between aMCI and naMCI at FDR-corrected *p* < 0.05

In naMCI, atrophy was mainly found in the triangular part of right inferior frontal gyrus compared to controls (Supplemental Table 1 and Fig. 1b), and the number of white matter fiber bundles passing through the triangular part of right inferior frontal gyrus were reduced (Supplemental Table 2 and Fig. 2b). The declines were concentrated at the proximal end of the center rather than the distal end of fibers passing from left to right when segmenting the fiber bundles (Supplemental Table 3 and Fig. 3), which may mainly involve the right anterior thalamic radiation. From the anterior to posterior direction, disruptions were focused at the front proximal end, mainly involving the right inferior fronto-occipital fasciculus. From the inferior to superior direction, declines were concentrated at the distal end of the center rather than the proximal end, mainly involving the right superior longitudinal fasciculus.

The total volume of clusters based on voxel-wise analysis was decreased in both MCI subtypes compared to controls, but there was no significant difference between the two MCI subtypes (Supplemental Table 2 and Fig. 1c, d). However, it is worth mentioning that the number of white matter fiber bundles passing through the triangular part of right inferior frontal gyrus was reduced in naMCI compared to aMCI (Supplemental Table 2 and Fig. 2d).

### Mediation analyses between atrophy, fiber bundles, and cognitions

The correlations between gray matter volume, white matter fiber bundle number, and cognitive scores are shown in Table 2. The gray matter volume of the left hippocampus was significantly related to MMSE, memory and language (|*r*| = 0.204–0.305, *all p* < 0.05), while fiber bundle number passing through the region was not related to any cognitive domain.

**Table 2:** Correlations between gray matter volume, fiber bundle number, and cognitive tests

	Gray matter volume		Fiber bundle number	
	Left hippocampus	Triangular part of right inferior frontal gyrus	Left hippocampus	Triangular part of right inferior frontal gyrus
MMSE	0.240^*^	0.140	0.100	0.052
**Memory**				
AVLT delay	0.305^**^	0.015	− 0.097	− 0.078
AVLT total	0.303^**^	0.017	− 0.060	− 0.009
R-O delay	0.274^**^	0.068	− 0.017	0.084
**Visual ability**				
R-O copy	0.022	0.198^*^	0.028	0.186^*^
CDT	0.005	0.074	0.113	0.140
**Language**				
CVFT	0.197^*^	0.112	0.086	0.080
BNT	0.281^**^	0.240^*^	0.143	0.125
**Attention**				
SDMT	0.157	0.222^*^	0.054	0.331^**^
TMTA	− 0.060	− 0.181^*^	− 0.059	− 0.286^**^
**Execution**				
StroopCtime	− 0.155	− 0.242^*^	− 0.162	− 0.196^*^
TMTB	− 0.101	− 0.195^*^	0.003	− 0.282^**^

*MMSE* Mini Mental Status Examination, *AVLT* Auditory Verbal Learning Test, *R-O* Rey-Osterrieth Complex Figure; *CDT*, Clock Drawing Test, *CVFT* Category Verbal Fluency Test, *BNT* Boston Naming Test, *SDMT* Symbol Digit Modalities Test, *TMT* Trail Making Test, *StroopCtime*, Time of Stroop Word Color Test C

^*^p < 0.05; **p < 0.001. Analyses were controlled for gender, education and age

In contrast, the gray matter volume of the triangular part of right inferior frontal gyrus was significantly related to all other cognitive domains, including visual ability, language, attention, and execution (|*r*| = 0.181–0.242, *all p* < 0.05), and fiber bundles passing through the region were significantly associated with visual ability, attention, and execution (|*r*| = 0.186–0.331, *all p* < 0.05).

**Fig. 4:**
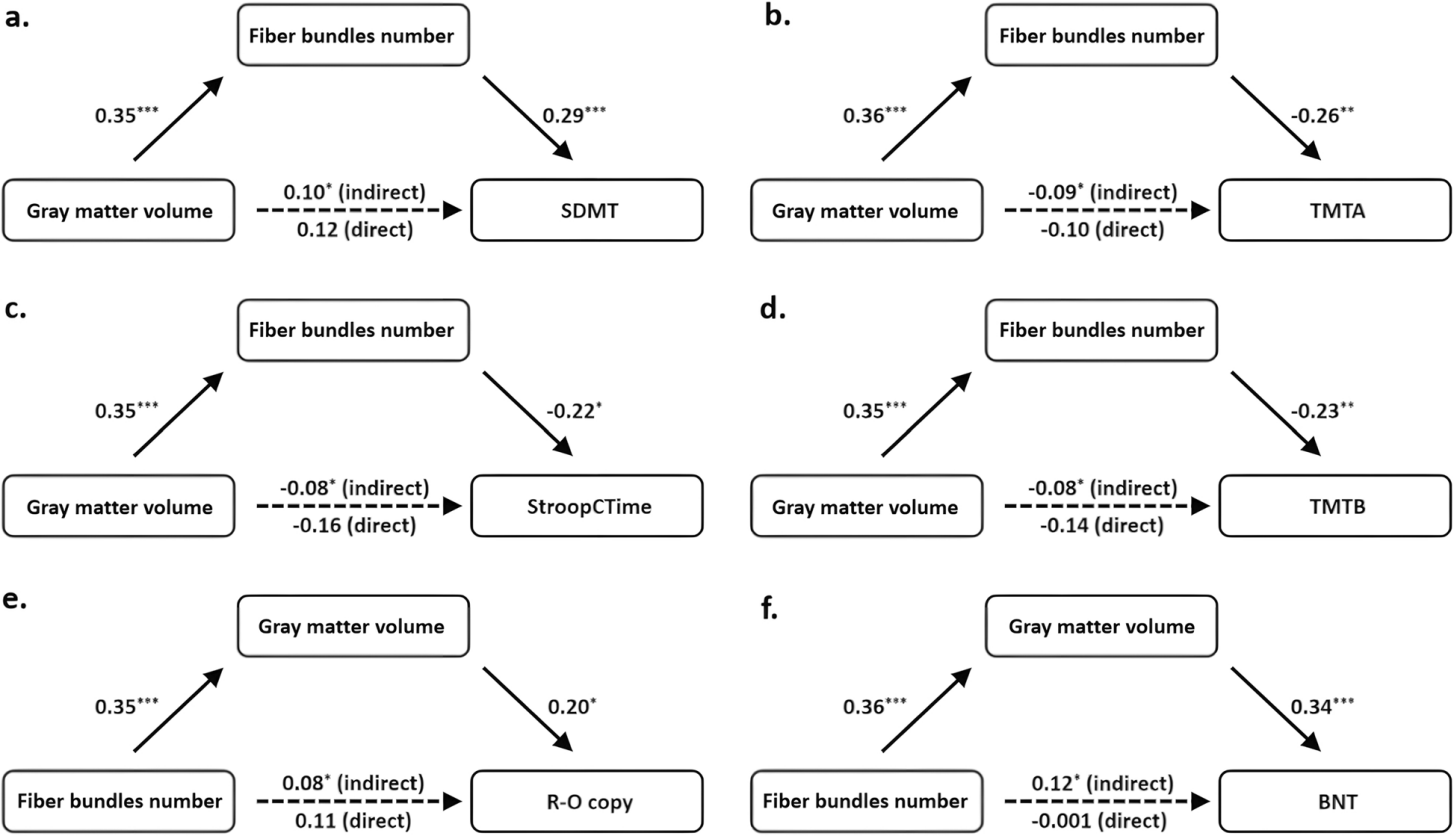
Mediation analysis of the relationship between fiber bundle number, gray matter volume in the triangular part of the right inferior frontal gyrus, and cognitive tests. The values are absolute standardized beta estimates of each mediation model. The significant relationships between gray matter volume and attention (**a**, **b**) and executive function (**c**, **d**) are mediated by fiber bundle number; the significant relationships between fiber bundle number and visual ability (**e**) and language (**f**) are mediated by gray matter volume

Based on the above correlation results, the brain indices of the triangular part of right inferior frontal gyrus and cognitive scores were included in the mediation analyses. The number of fiber bundles significantly mediated the relationships between gray matter volume and attention and execution, while gray matter volume significantly mediated the relationships between fiber bundle number and visual ability and language (Supplemental Table 4 and Fig. 4).

## Discussion

The study aimed to uncover the divergent structural damage patterns of aMCI and naMCI and their correlation with impaired cognition. Results indicated that the structural damage in aMCI primarily affected the volume of the hippocampus and was related to memory, overall cognitive ability, and language. However, analysis of naMCI showed gray matter atrophy in the inferior frontal gyrus, with significant damage to fiber bundles traversing the region in all three directions, not only affecting fibers around the atrophied area but also distant fibers. It was observed that the number of fiber bundles in this frontal region had a closer connection with execution and attention, while the volume atrophy was more closely linked to language and visual ability.

The association between the hippocampus and memory has been extensively researched and proven. This brain region often experiences atrophy in memory-related neurodegenerative diseases, such as AD and aMCI [21]. Indeed, the present study found that individuals with aMCI had significant atrophy in the volume of hippocampus compared to control group, and this atrophy accompanied by declines in memory. Unlike some previous studies [22], this study did not find any other structural changes in the brains of aMCI individuals, such as atrophy of other brain regions or white matter. The possible two reasons for these differences may be attributed to the definition of aMCI and the indicators used. First, it is related to the aMCI definition. Some studies [23] have included patients with memory impairment and other cognitive impairments as aMCI. The brain structural differences explored may thus represent the impairment of other cognitive functions; second, it is related to the brain indicators used in different studies. A previous study found decreased cortical thickness in the precuneus region of aMCI individuals [22]. However, other studies have only found gray matter atrophy (but not white matter) in the hippocampus [24]. A recent meta-analysis [21] of 31 studies found that changes in the hippocampus and surrounding regions (such as the parahippocampus and amygdala, which was also examined in this study) were highly reproducible, while no changes in other brain regions were reproducible.

The present study found that naMCI individuals had significant atrophy of gray matter in the right inferior frontal gyrus, which is related to various cognitive functions, including execution [25], attention [25], language [26], and visual ability [27]. The damage to fiber bundles in this area mainly involved the right inferior fronto-occipital fasciculus, the anterior thalamic radiation, and the superior longitudinal fasciculus. The right inferior fronto-occipital fasciculus, a bunch of long complex fiber bundles connecting the orbitofrontal cortex to the occipital lobe, is thought to participate in reading, attention, executive function, and visual processing [28]. The anterior thalamic radiation with nerve fibers serves as part of the fronto-striato-thalamic loop and provides important contributions to executive function [29]. The superior longitudinal fasciculus, with an obvious endpoint in the triangular part of the inferior frontal gyri, plays a role in both attention and language [30]. The disconnections of these complex fiber bundles passing through the right inferior frontal gyrus may jointly explain various cognitive disorders in the naMCI population. It is worth noting that asymmetric loss of these fiber bundles in different directions may indicate different patterns of fiber damage, with the superior longitudinal fasciculus possibly being damaged at its distal end, and the remaining fiber bundles at the proximal end.

The fiber bundles were found to mediate the relationships between gray matter volume and attention and executive function. Other studies have determined that white matter hyperintensities better characterize these cognitive functions in elderly individuals without cognitive impairment than gray matter volume [31]. Moreover, studies on neurodegenerative, including FTD, have found that white matter disruption is a significant factor in executive function impairment [7]. Studies have shown that the integrity of white matter fibers connecting the frontal lobe impacts executive function [32]; indeed, we observed the disconnection of fibers near the gray matter region [29]. These cognitive functions that depend on networks may require increased connectivity with different regions, provided by additional fiber bundles, for normal functioning such as the fronto-parietal network and ventral attention network [33].

Some studies have shown that gray matter played as a more influential factor than white matter to affect cognition, particularly memory [34], and in memory-impaired participants, such as AD [35]. This pattern suggests that different cognitive functions may have distinct closely associated pathways. Indeed, visual ability and language were more closely related to gray matter than white matter. The structure of the occipital region, which is far from the frontal lobe, may also play a role in visual ability [33]. Our fiber segmentation analysis indicated that the decline in spatial ability observed in MCI patients was not due to a decrease in posterior distal fiber bundles, suggesting that white matter damage was not the cause. A significant decline in language was also found in the amnestic MCI group, suggesting that language is not solely dependent on the frontal lobe. The inferior frontal gyrus is considered an important area for language production [36], but the language tasks in this study were relatively simple and directly related to language production, so performance may be more related to the functions of specific brain regions than the connections between networks, such as the temporal lobe, which is crucial for language comprehension.

One limitation of the study is that the sample population of naMCI consisted of patients with multiple cognitive impairments. In this study, there were relatively fewer individuals with single-domain naMCI, especially within the domains of language and visual abilities. The study was unable to investigate the characteristics of individuals who had specific cognitive impairments. As a result, the brain regions that were found to be atrophied in this study may not be the main regions responsible for these cognitions. Precision medicine requires the accurate classification of patients and a detailed description of the corresponding pathological characteristics. Despite this limitation, the study found strong correlations between these cognitive functions and brain indices, which future studies with larger sample sizes with specific cognitive impairment can test and verify.

## Conclusion

The aMCI was characterized by atrophy of the left hippocampal volume, while the naMCI exhibited both volumetric atrophy and disconnection of fiber bundles traversing the right inferior frontal gyrus, affecting both long-range and short-range fibers. The divergent structural damage patterns result in the emergence of different subtypes of MCI, leading to a decline in corresponding cognitive function. These findings suggest that accurately detecting patients with diverse cognitive impairments in the early stages of neurodegenerative diseases can deepen our understanding of dementia subtypes or other severe degenerative diseases and offer potential avenues for early clinical interventions.

### Supplementary Information

**Additional file 1:** **Supplemental Table 1.** The voxel-wise gray matter regions with significant difference between groups. **Supplemental Table 2.** Difference in the gray matter volume and fiber bundle number between groups. **Supplemental Table 3.** The differences in each segment of fiber bundles between groups. **Supplemental Table 4.** Mediating model results of volume, fiber bundle number and cognitions. **Supplemental Fig. 1.** Flowchart shows the selection criteria and the number of participants enrolled for various MCI groups. SD-aMCI, single-domain aMCI; MD-aMCI, multi-domain aMCI. **Supplemental Fig. 2.** Group differences of each fiber bundle segment number based on the seed of the left hippocampus along X, Y, Z axis (MNI coordinates). The central point of the seed in the X, Y, Z axis direction fell on the seventh, the seventh, and the tenth segment, separately.

## Data Availability

Data and code used in this study can be made available after a reasonable request to the authors following a formal data sharing agreement.

## Abbreviations

aMCI: Amnestic mild cognitive impairment
naMCI: Non-amnestic mild cognitive impairment
AD: Alzheimer’s disease
FTD: Frontotemporal dementia
MMSE: Mini-Mental Status Examination
AVLT: Auditory Verbal Learning Test
R-O: Rey-Osterrieth Complex Figure
CDT: Clock-Drawing Test
CVFT: Category Verbal Fluency Test
BNT: Boston Naming Test
SDMT: Symbol Digit Modalities Test
TMT: Trail-Making Test
TIV: Total intracranial volume
