# Supplementary material for: Divergent brain regional atrophy and associated fiber disruption in amnestic and non-amnestic MCI
Source: Alzheimers Res Ther. 2023 Nov 13;15:199. doi: 10.1186/s13195-023-01335-1 (PMC10642051; doi:10.1186/s13195-023-01335-1)
Supplement: Supplementary file 1 — Additional file 1: Supplemental Table 1. The voxel-wise gray matter regions with significant difference between groups. Supplemental Table 2. Difference in the gray matter volume and fiber bundle number between groups. Supplemental Table 3. The differences in each segment of fiber bundles between groups. Supplemental Table 4. Mediating model results of volume, fiber bundle number and cognitions. Supplemental Fig. 1. Flowchart shows the selection criteria and the number of participants enrolled for various MCI groups. SD-aMCI, single-domain aMCI; MD-aMCI, multi-domain aMCI. Supplemental Fig. 2. Group differences of each fiber bundle segment number based on the seed of the left hippocampus along X, Y, Z axis (MNI coordinates). The central point of the seed in the X, Y, Z axis direction fell on the seventh, the seventh, and the tenth segment, separately. [file 13195_2023_1335_MOESM1_ESM.docx]

**Supplemental Table 1** The voxel-wise gray matter regions with significant difference between groups.

|  | Peak MNI (x y z) | | | Voxels | Peak intensity |
| --- | --- | --- | --- | --- | --- |
| controls > aMCI | | | | | |
| left hippocampus | -28.5 | -12 | -18 | 1263 | 4.54 |
| controls > naMCI | | | | | |
| triangular part of right inferior frontal gyrus | 55.5 | 27 | 3 | 932 | 4.67 |

The significance level was set at a voxel-level inference of *p* < 0.001 combined with a cluster-level inference of *p* < 0.05 (GRF-corrected). Analyses were corrected for demographic variables and TIV.**Supplemental Table 2** Difference in the gray matter volume and fiber bundles number between groups.

|  | controls | aMCI | naMCI | *F* | *p* |
| --- | --- | --- | --- | --- | --- |
| gray matter volume (mm^3^) | | | | | |
| left hippocampus | 2192±32 | 1998±30 | 2068±31 | 10.3 | < 0.001^a,b^ |
| triangular part of right inferior frontal gyrus | 208±5 | 193±5 | 179±5 | 7.5 | < 0.001^a,b^ |
| fiber bundles number | | | | | |
| left hippocampus | 9968±4832 | 10823±6132 | 9037±5307 | 1.3 | 0.270 |
| triangular part of right inferior frontal gyrus | 5275±3355 | 4660±3450 | 3163±3170 | 5.7 | < 0.01^b,c^ |

Analyses were controlled for gender, education and age.

^a^Statistically significant difference between controls and aMCI at *p* < 0.05.

^b^Statistically significant difference between controls and naMCI at *p* < 0.05.

^c^Statistically significant difference between aMCI and naMCI at *p* < 0.05.**Supplemental Table 3** The differences in each segment of fiber bundles between groups.

| *F* | Left hippocampus | | | triangular part of right inferior frontal gyrus | | |
| --- | --- | --- | --- | --- | --- | --- |
|  | X axis | Y axis | Z axis | X axis | Y axis | Z axis |
| Segment 1 | - | 1.0 | 0.2 | - | 1 | - |
| Segment 2 | - | 1.8 | 0.1 | - | 0.7 | 0.9 |
| Segment 3 | - | 3.2 | 1.7 | 1 | 1.4 | 0.6 |
| Segment 4 | 1.4 | 1.5 | 0.9 | 0.4 | 2.8b | 1.8b |
| Segment 5 | 0.2 | 2.3 | 0.5 | 0.3 | 8.4b,c | 3.2b |
| Segment 6 | 0.0 | 1.3 | 0.1 | 0.1 | 1.5 | 2.6 |
| Segment 7 | 0.3 | 0.3 | 0.8 | 0.1 | 2.6 | 1.2 |
| Segment 8 | 1.9 | 0.5 | 0.9 | 0.1 | 3.6 | 4.1b |
| Segment 9 | 0.9 | 2.5 | 0.6 | 1.4 | 2.8 | 4.4b |
| Segment 10 | 2.0 | 2.7 | 0.4 | 7.5b,c | 2.6 | 2.2b |
| Segment 11 | 2.0 | 2.5 | 1.0 | 8.7b,c | 1.6 | 1.5 |
| Segment 12 | 3.5 | 1.2 | - | 4.9b,c+ | 1.3 | - |
| Segment 13 | 1.1 | 0.6 | - | 0.2 | 0.1 | - |
| Segment 14 | 0.8 | 0.6 | - | - | 0.2 | - |

Analyses were controlled for gender, education and age.

^b^Statistically significant difference between controls and naMCI at FDR-corrected *p* < 0.05.

^c^Statistically significant difference between aMCI and naMCI at FDR-corrected *p* < 0.05.

^c+^Statistically marginally significant difference between aMCI and naMCI at FDR-corrected *p* < 0.05.**Supplemental Table 4** Mediating model results of volume, fiber bundles number and cognitions.

| Model | Bootstrap estimates for a×b |
| --- | --- |
| A.1: gray matter-fiber bundles-R-O copy | -0.0268, 0.1007 |
| A.2: fiber bundles-gray matter-R-O copy | 0.0239, 0.1368 |
| B.1: gray matter-fiber bundles-SDMT | 0.0256, 0.1857 |
| B.2: fiber bundles-gray matter-SDMT | -0.0124, 0.1136 |
| C.1: gray matter-fiber bundles-TMTA | -0.1628, -0.0352 |
| C.2: fiber bundles-gray matter-TMTA | -0.0948, 0.0118 |
| D.1: gray matter-fiber bundles-StroopCTime | -0.1192, -0.0042 |
| D.2: fiber bundles-gray matter-StroopCTime | -0.1124, 0.0128 |
| E.1: gray matter-fiber bundles-TMTB | -0.1345, -0.0329 |
| E.2: fiber bundles-gray matter-TMTB | -0.1249, 0.0053 |
| F.1: gray matter-fiber bundles-BNT | -0.0588, 0.0553 |
| F.2: fiber bundles-gray matter-BNT | 0.0564, 0.2046 |

The indirect effect is significant when the Bootstrap estimates for the beta distribution does not contain 0.

R-O, Rey-Osterrrieth Complex Figure; SDMT, Symbol Digit Modalities Test; TMT, Trail Making Test; StroopCtime, Time of Stroop Word Color Test C; BNT, Boston Naming Test.


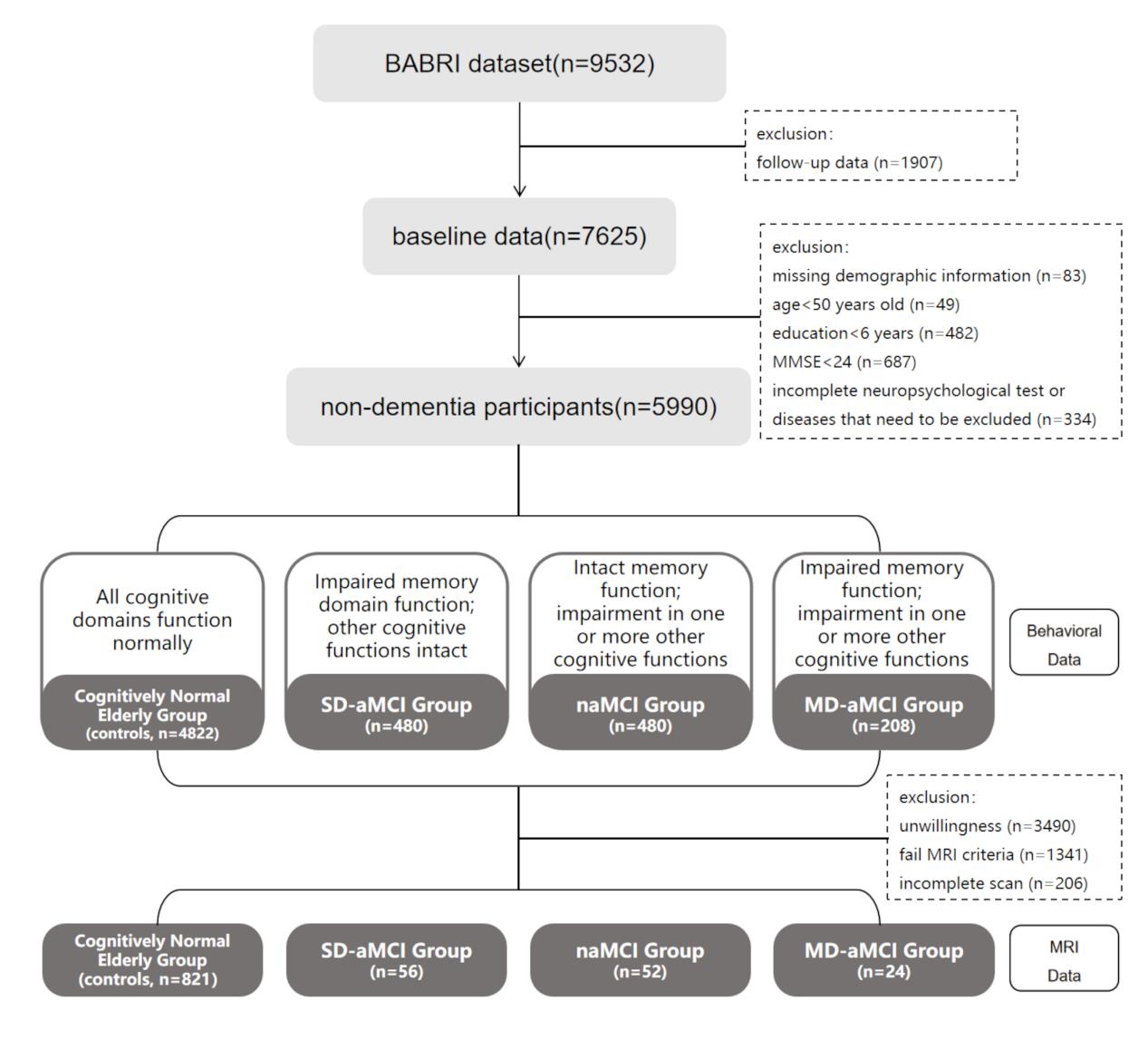


**Supplemental Fig. 1** Flowchart shows the selection criteria and the number of participants enrolled for various MCI groups. SD-aMCI, single-domain aMCI; MD-aMCI, multi-domain aMCI.

**
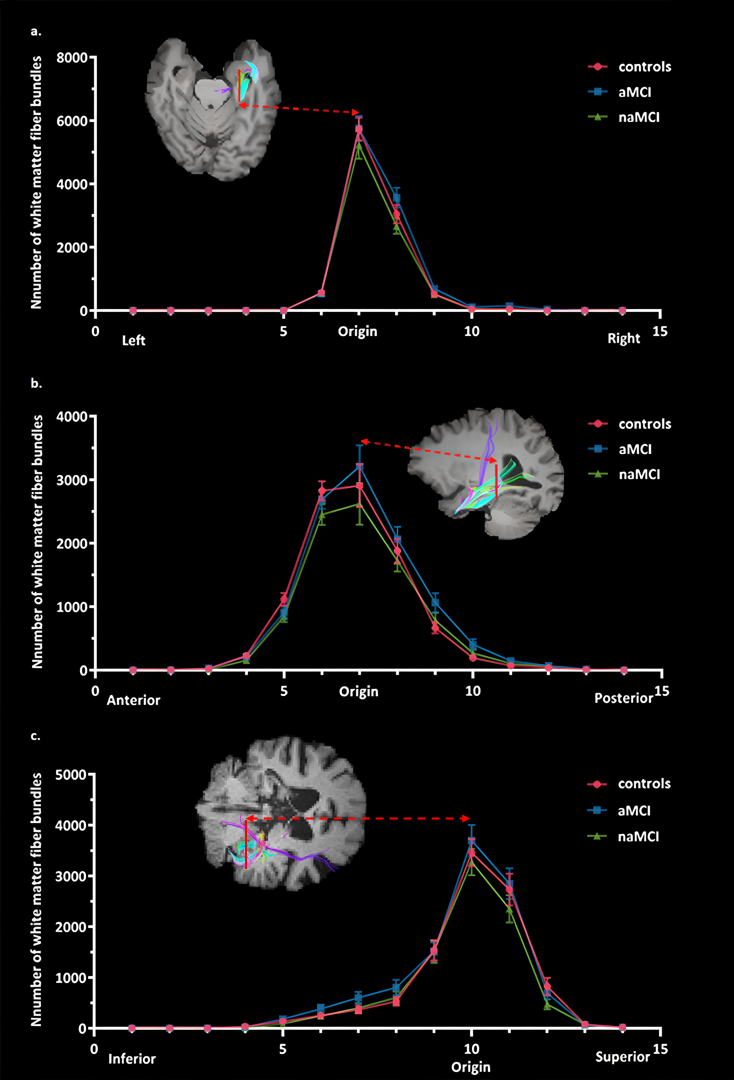
**

**Supplemental Fig. 2** Group differences of each fiber bundles segment number based on the seed of the left hippocampus along X, Y, Z axis (MNI coordinates). The central point of the seed in the X, Y, Z axis direction fell on the seventh, the seventh, and the tenth segment, separately.
